# Supplementary material for: Genetic Attenuation of Paraoxonase 1 Activity Induces Proatherogenic Changes in Plasma Proteomes of Mice and Humans
Source: Antioxidants (Basel). 2020 Nov 28;9(12):1198. doi: 10.3390/antiox9121198 (PMC7761039; doi:10.3390/antiox9121198)
Supplement: Supplementary file 1 [file antioxidants-09-01198-s001.pdf]

Supplementary material

Supplementary Table S1.

Supplementary Table S2.

Supplementary Table S1. Characteristics of participants.

**Variable**	**All (n=100)**	***PON1-192QQ* (n = 51)**	***PON1-192QR* (n = 30)**	***PON1-192RR* (n = 19)**	**Annova *p*-value**
Female sex, n (%)	50 (50)	26 (51)	14 (47)	10 (53)	
Age, years	48.9±16.8	49.7±18.8	49.6±13.8	45.7±14.5	0.654
Glucose, mM	5.7±0.8	5.6±0.9	5.8±0.7	5. 7±0.82	0.687
T-C, mg/dl	202.5±41.5	192.9±36.0	211,0±33.2	214.7±59.9	0.059
HDL-C, mg/dl	63.4±18.3	61.2±16.1	68.9±20.7	60.4±19.1	0.139
LDL-C, mg/dl	115.1±37.2	109.0±33.2	117.3±35.1	127.9±47.6	0.159
TG, mg/dl	123.4±90.0	114.6±87.1	132.4±110.7	132.7±57.8	0.615
Creatinine, μM	73.9±113.0	73.1±12.8	74.8±13.1	74.6±13.8	0.825
e-GFR, ml/min/pc	85.6±8.7	85.6±9.1	85.5±8.8	85.8±8.2	0.994
GSH, μM	3.9±3.0	3.8±3.2	4.2±3.1	3.9±2.6	0.860
Hcy, μM	5.7±2.1	5.5±2.1	6.0±2.1	6.0±2.1	0.499
Cys, μM	215.4±67.7	217.2±74.5	210.0±63.1	219.1±57.7	0.869
CysGly, μM	18.7±11.7	18.2±11.9	19.0±11.9	19.7±11.5	0.884
POase, units	0.011±0.009	0.0033±0.0034	0.016±0.002	0.023±0.005	0.000
PhAcase, units	0.455±0.111	0.396±0.070	0.546±0.103	0.469±0.113	0.000

T-C, Total cholesterol; HDL-C, high-density lipoprotein cholesterol; LDL-C, low-density lipoprotein cholesterol; TG, triglycerides; e-GFR, estimated glomerular filtration rate; GSH, glutatione; Hcy, homocysteine; Cys, cysteine; CysGly, cysteinylglycine; POase, PON1 activity assayed with paraoxon (Jakubowski, 2000); PhAcase, PON1 activity assayed with phenyl acetate (Jakubowski, 2000).

Supplementary Table S2. *PON1* genotype-responsive proteins in humans and mice.

Gene Name	Protein ID	Protein Name	*PON1 192QQ vs. RR+QR*		*PON1 192QQ vs. QR*		*PON1 192QR vs. RR*		*PON1 192QQ vs. RR*		ANOVA	MOUSE *Pon1*$^{-/-}$ *vs. Pon1*$^{+/+}$		Molecular Function/Biological Process
			Fold change	*p* value	Fold change	*p* value	Fold change	*p* value	Fold change	*p* value	*p* value	Fold change	*p* value	
Afm	O89020	Afamin										0.77	0.008	Vitamin transport
N/A	Q96SB0	Anti-streptococcal/anti-myosin Ig λ light chain var. reg. region	0.81	0.016	0.80	0.030								Immune response
Serpinc1	P32261	Antithrombin-III										0.85	0.032	Complement and coagulation cascades
APOA1	Q00623	Apolipoprotein A-I			0.88	0.033						0.87	0.041	Fat digestion/absorption
Apob	E9Q414	Apolipoprotein B-100										1.43	0.003	Cholesterol homeostasis; Fat digestion/absorption
Apoc1	P34928	Apolipoprotein C-I										0.64	0.041	Lipid transport/metabolism
Apod	P51910	Apolipoprotein D					1.15	0.045				1.19	0.044	Aging; Brain development
Apom	Q9Z1R3	Apolipoprotein M			0.88	0.008	1.15	0.031			0.021	1.28	0.002	Cholesterol transport
Bpgm	P15327	Bisphosphoglycerate mutase										0.30	0.010	Erythrocyte development; Glycolysis
Clu	Q06890	Clusterin										1.14	0.047	Chaperone-mediated protein folding; immune complex clearance; inhibits amyloid formation
F13b	Q07968	Coagulation factor XIII B chain							0.90	0.046		0.70	0.011	Blood coagulation; fibrin-stabilizing factor; negative regul. of cell protein catabolic process
C9	A0A024R035	Complement component C9	1.13	0.011	1.16	0.007					0.024			Complement/coagulation cascades
Cfh	E9Q8I0	Complement factor H										1.24	2E-04	Complement/coagulation cascades
AI182371	A2AS37	Expressed sequence AI182371										1.23	0.047	Complement activation, inflammatory response
FETUB	Q9QXC1	Fetuin-B	0.76	0.013								1.18	0.005	Egg fertilization; Sperm binding; Inhibits Cys-endopeptidase activity
Blvrb	Q923D2	Flavin reductase (NADPH)										0.44	0.042	Heme catabolic process
Aldoa	A6ZI44	Fructose-bisphosphate aldolaseA										0.45	0.004	ATP biosynthetic process

GPX3	A0A087X1J7	Glutathione peroxidase 3			0.78	0.029	1.77	5E-05			0.0014			Cellular response to oxidative stress
Hp	Q61646	Haptoglobin			0.83	0.022						4.50	0.037	Acute phase response
Hpx	Q91X72	Hemopexin										1.18	0.002	Cellular iron ion homeostasis
Hgfac	Q9R098	Hepatocyte growth factor activator										0.79	0.008	Serine-type endopeptidase activity
Hrg	Q9ESB3	Histidine-rich glycoprotein										1.17	0.005	Angiogenesis, Fibrinolysis
Igha	A0A0A6YX W6	Ig alpha chain C region										2.92	4E-06	Immune response
Ighg1	P01868	Ig gamma-1 chain C region secreted form										1.64	0.022	Immune response
Ighg1	P01869	Ig gamma-1 chain C region secreted form										2.31	2E-04	Immune response
Ighg3	P03987	Ig gamma-3 chain C region							1.30	0.015		0.32	1E-04	Immune response
Ighv3-6	P18531	Ig heavy chain V region 3-6										1.65	0.033	Immune response
Ighv7-1	P01790	Ig heavy chain V region M511										2.14	0.013	Immune response
N/A	P01638	Ig kappa chain V-V region L6										1.58	0.001	Immune response
Gm5571	P01639	Ig kappa chain V-V region MOPC 41										1.58	0.014	Immune response
V2-17	Q5NV90	V2-17 protein					1.23	0.042						Immune response
N/A	P01843	Ig λ-1 chain C region										1.52	0.010	Immune response
Iglv1	P01723	Ig λ-1 chain V region										2.64	0.002	Immune response
Ighm	A0A075B6 A0	Ig mu chain C region										1.59	0.002	Immune response
Ighv10-1	A0A0B4J1J6	Ig heavy variable 10-1										1.76	0.026	Immune response
Ighv1-76	A0A0G2JFE9	Ig heavy variable 1-76										1.61	0.025	Immune response
Igj	P01592	Immunoglobulin J chain										2.99	1E-05	Immune response
Igkv19-93	A0A0G2JFZ3	Ig kappa variable 19-93										2.26	0.019	Immune response
Igkv17-127	A0A0G2JDN5	Ig kappa variable 17-127										2.85	0.006	Immune response
Igkv4-63	A0A0G2JFU6	Ig kappa variable 4-63										1.89	0.040	Immune response
Igkv8-28	A0A0G2JE47	Ig kappa variable 8-28										3.53	0.001	Immune response
Ica	Q9DBD0	Inhibitor of carbonic anhydrase										0.89	0.004	Enzyme inhibitor activity
Itih1	Q61702	Inter-α-trypsin inhi-bitor heavy chain H1										0.90	0.002	Endopeptidase inhibitor
ITIH3	Q06033	Inter-α-trypsin inhi-bitor heavy chain H3	1.30	0.001	1.22	0.023			1.45	7E-05	0.0008			Endopeptidase inhibitor

Lifr	P42703	Leukemia inhibitory factor receptor										0.72	3E-04	Cytokine-mediated signaling
Ldha	P06151	L-lactate dehydro-genase A chain										0.48	4E-04	Cellular response to extracellular stimulus
Mbl1	P39039	Mannose-binding protein A										0.83	0.045	Complement/coagulation cascades; Phagosome
Mug1	P28665	Murinoglobulin-1										0.77	0.032	Embryo implantation
PGLYRP2	Q96PD5	N-acetylmuramoyl-L-alanine amidase	1.08	0.013	1.09	0.021					0.047			Antimicrobial humoral response
Lcat	P16301	Phosphatidylcholine-sterol acyltransferase										1.41	0.036	Plasma lipoprotein metabolism
Klkb1	P26262	Plasma kallikrein										0.83	0.015	Complement/coagulation cascades
PLG	P00747	Plasminogen	0.95	0.048										Blood coagulation
CFP	P27918	Properdin	1.32	0.013	1.34	0.034								Complement activation, immune response
Ambp	Q07456	Alpha-1-microglobulin										1.15	0.011	Protein catabolic process; Inhibits trypsin, plasmin, elastase
SERPINA10	Q9UK55	Protein Z-dependent protease inhibitor					0.78	0.026	0.78	0.021	0.034			Blood coagulation
F2	Q8R121	Prothrombin										1.11	0.022	Complement/coagulation cascades
RBP4	P02753	Retinol-binding protein 4	0.88	0.002	0.89	0.016			0.86	0.009	0.006			Cardiac muscle tissue development
Alb	P07724	Serum albumin										0.88	0.036	Cellular response to starvation
Pon1	P52430	Serum paraoxonase 1	0.62	4E-16	0.63	6E-12			0.60	5E-11	3E-15	0.02	3E-19	Homocysteine thiolactone detoxication
Ttr	P02766	Transthyretin	0.84	0.007	0.83	0.012					0.022	1.28	0.037	Retinol metabolic process, thyroid hormone transport
V2-6	A2MYD5	V2-6 protein			1.27	0.038								Immune response
VTN	P04004	Vitronectin					0.91	0.007						Collagen binding, complement/cogulation cascades

[a] Proteins affected by *PON1* genotype only in humans or mice are highlighted in yellow or bright green, respectively. Data for proteins affected by *PON1* genotype in both humans and mice are shaded in green accent light.
